# Activation Energy of Aggregation-Disaggregation Self-Oscillation of Polymer Chain

**DOI:** 10.3390/ijms131216281

**Published:** 2012-12-03

**Authors:** Yusuke Hara, Rumana A. Jahan

**Affiliations:** Nanosystem Research Institute, NRI, National Institute of Advanced Science and Technology, AIST, Central 5-2, 1-1-1 Higashi, Tsukuba 305-8565, Japan; E-Mail: rumanafi@yahoo.com

**Keywords:** self-oscillation, polymer chain, BZ reaction, molecular robot

## Abstract

In this paper, we investigated the activation energies of the aggregation–disaggregation self-oscillation induced by the Belousov-Zhabotinsky (BZ) reaction by utilizing the nonthermoresponsive polymer chain in a wide temperature range. This is because the conventional type self-oscillating polymer chain, with thermoresponsive poly(Nisopropylacrylamide) (poly(NIPAAm) main-chain covalently bonded to the ruthenium catalyst (Ru(bpy)_3_) of the BZ reaction, cannot evaluate the activation energy over the lower critical solution temperature (LCST). The nonthermoresponsive self-oscillating polymer chain is composed of a poly-vinylpyrrolidone (PVP) main-chain with the ruthenium catalyst (Ru(bpy)_3_). As a result, we clarified that the activation energy of the aggregation–disaggregation self-oscillation of the polymer chain is hardly affected by the concentrations of the BZ substrates. In addition, the activation energy of the nonthermoresponsive self-oscillating polymer chain was found to be almost the same value as normal BZ reaction, *i.e.*, not including the self-oscillating polymer system with Ru moiety.

## 1. Introduction

**Figure 1:**
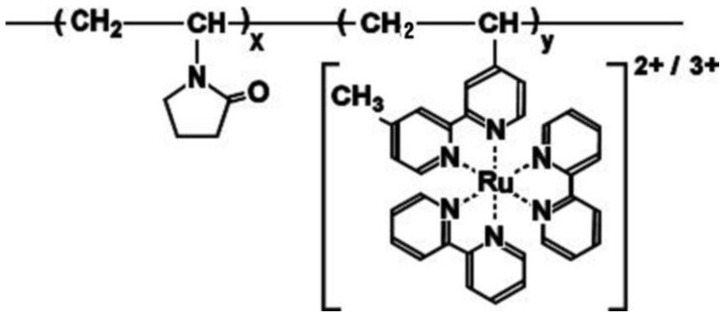
Chemical structure of the nonthermoresponsive self-oscillating polymer chain.

The Belousov-Zhabotinsky (BZ) reaction has been experimentally and theoretically studied because the BZ reaction can be treated as a simple model for the formation of a spatiotemporal structure, such as spiral pattern or target pattern in an unstirred solution, and multistability, periodicity, multiperiodicity in a stirred solution [1–8]. The overall process of the BZ reaction is the oxidization of an organic substrate by an oxidizing agent in the presence of the catalyst under strong acidic conditions. In the BZ reaction, the oxidation number of the ruthenium tris(2,2′-bipyridine), *i.e.*, the metal catalyst of the BZ reaction, causes periodical change. At the same time, the solubility of the Ru catalyst changes periodically. In previous studies, in order to develop the autonomous polymer system, the polymer chains covalently bonded to the Ru catalyst were synthesized [9,10]. As a result, the polymer chains cause the aggregation–disaggregation self-oscillation under the constant temperature condition induced by the BZ reaction. The aggregation–disaggregation self-oscillation originates from the periodical solubility change of the Ru moiety in the polymer chain. Moreover, by introducing the cross-linker into the self-oscillating polymer chain, polymer gel with the Ru(bpy)_3_ catalyst was synthesized [11]. The polymer gel causes swelling–deswelling self-oscillations under the constant temperature condition induced by the BZ reaction. Recently, the self-oscillating polymer system with a negatively charged acrylamide-2-methylpropanesulfonic acid (AMPS) as a pH and a solubility control site was developed in order to expand the autonomous behavior [12–20]. By utilizing the AMPS-containing self-oscillating polymer system, novel phenomena such as an on-off switching of the aggregation–disaggregation self-oscillation and a viscosity self-oscillation of the high concentration of the AMPS-containing polymer solutions were observed [15,16]. Moreover, the displacement of the self-oscillating polymer gel with the AMPS moiety significantly increases compared with the conventional-type self-oscillating poly(NIPAAm-*co*-Ru(bpy)_3_) gel [11,17–20]. By utilizing the large displacement of the AMPS-containing polymer gel, a self-walking gel robot and a matter transport gel system were first developed. However, these self-oscillating polymer systems have a temperature limitation because the main-chain of the polymer system was composed of thermoresponsive poly(Nisopropylacrylamide) (poly(NIPAAm)). Therefore, the application field was restricted by the temperature limitation. If we could cause the self-oscillation under high temperature condition, we can construct the autonomous polymer actuators with high frequency motion. That is because the motion speed of the self-oscillating polymer system depends on the temperature. In order to expand the temperature condition, the self-oscillating polymer chain with a nonthermoresponsive nature was required, as the nonthermoresponsive self-oscillating polymer system can cause the self-oscillation in the wide temperature conditions. In our previous study, a nonthermoresponsive poly-vinylpyrrolidone (PVP) was selected as the self-oscillating polymer main-chain (see Figure 1). The influence of initial substrate concentrations of the BZ reaction on the transmittance self-oscillating behavior was investigated at only 14 °C [21]. Moreover, by utilizing the VP-based polymer chain with the different Ru(bpy)_3_ content, we evaluated the influence of the polymer concentration, the BZ substrates conditions and the temperature on the self-oscillating behavior [22,23].

In this study, we evaluated the activation energy of the aggregation–disaggregation self-oscillation in broad concentrations of the three BZ substrates in a wide temperature range. That is because the thermoresponsive self-oscillating polymer system cannot evaluate the activation energy in the wide temperature condition due to the lower critical solution temperature (LCST). As a result of the evaluation of the oscillation period in the wide temperature range, we demonstrated that the activation energy of the self-oscillating polymer chain is hardly affected by the concentrations of the three BZ substrates. In addition, the activation energy is almost the same value as the conventional type self-oscillating gel that was evaluated only below the LCST due to the thermoreponsive nature. Furthermore, the activation energy of the self-oscillating polymer chain has almost the same value as the normal BZ reaction in aqueous solution, *i.e.*, not including the self-oscillating polymer system.

## 2. Results and Discussion

**Figure 2:**
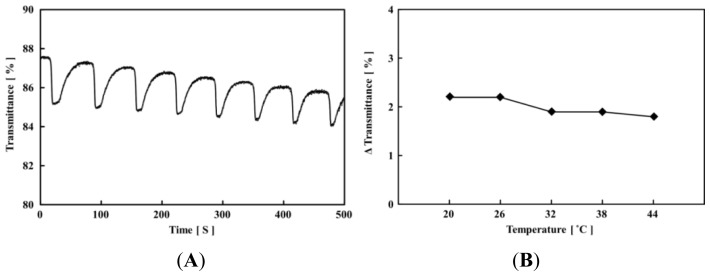
(**A**) Oscillating profiles of the transmittance for the 0.5 wt% polymer solutions containing fixed initial concentrations of the Belousov-Zhabotinsky (BZ substrates ([MA] = 0.1 M, [HNO_3_] = 0.3 M and [NaBrO_3_] = 0.3 M) at 20 °C. (**B**) Dependence of amplitude of transmittance self-oscillation at different temperature conditions (*T* = 20, 26, 32, 38, 44 °C).

Figure 2A shows self-oscillating behaviors of the poly(VP-*co*-Ru(bpy)_3_) solution at 20 °C under the fixed concentrations of the three substrates of the BZ reaction ([MA] = 0.1 M, [NaBrO_3_] = 0.3 M and [HNO_3_] = 0.3 M). The transmittance self-oscillation originates from the different solubility of the VP-based polymer chain with the Ru(bpy)_3_ moiety in the reduced and oxidized state. In our previous investigation, we conducted the measurement of the LCST for the VP-based self-oscillating polymer chain [21,22]. As a result, it was clarified that there are no LCST in the reduced and oxidized state due to the nonthermorepsponsive nature of the polymer main-chain. Moreover, the LCST measurement demonstrated that the polymer chain in the reduced and oxidized state has the different solubility. The different solubility of the polymer chain in the reduced and oxidized state works as the driving force of the transmittance self-oscillation. In addition, Figure 2B shows the amplitude of the transmittance self-oscillation in the same concentration of the three BZ substrates at the different temperature conditions (20–44 °C). As shown in Figure 2B, the amplitude of the transmittance self-oscillation for the self-oscillating polymer chain is hardly affected by the temperature. This tendency is different from the NIPAAm-based polymer chains because the aggregation state of the thermoresponsive polymer chain is much affected by the temperature [12–15]. In the self-oscillating behavior, the polymer aggregation state changes with time because the polymer aggregation state depends on the solubility of the polymer chain. As for the VP-based polymer chain, the aggregation state is hardly affected by the temperature due to the nonthermoresponsive nature. Therefore, the amplitude of the VP-based self-oscillating polymer chain is hardly affected by the temperature.

**Figure 3:**
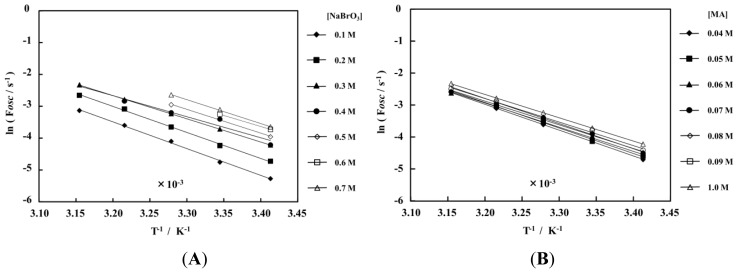

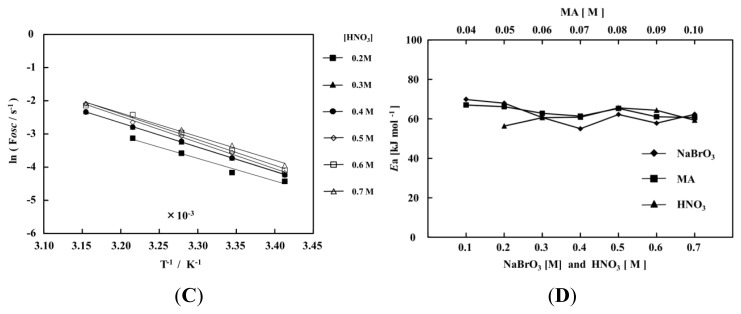
Arrenius dependence on temperature of oscillating frequency, F*osc*, under fixed concentrations of the other two BZ substrates: (**A**) [MA] = 0.1 M and [HNO_3_] = 0.3 M; (**B**) [NaBrO_3_] = 0.3 M and [HNO_3_] = 0.3 M; (**C**) [NaBrO_3_] = 0.3 M and [MA] = 0.1 M. (**D**) Dependence of activation energy on concentrations of three BZ substrates.

Figure 3A–C shows the Arrhenius dependence on the temperature (20–44 °C) in the wide concentration rage of the three BZ substrates. As shown in Figure 3A–C, all plots have a good linear relationship. As shown in Figure 3A–C, the period of the self-oscillation for the polymer chain has the same temperature dependence in all the BZ substrates conditions. Figure 3D shows the dependence of activation energies of the self-oscillating polymer chain. As shown in Figure 3D, the activation energy is hardly affected by the concentrations of the three BZ substrates. In addition, the activation energy is almost the same value as the conventional-type self-oscillating poly(NIPAAm-co-Ru(bpy)_3_) gel below the LCST [24]. Moreover, the activation energy of the self-oscillating polymer system is almost the same value as the normal BZ reaction, *i.e.*, not including the self-oscillating polymer system with the Ru moiety [25].

In Table 1, we summarized the self-oscillating region given by the initial concentrations of the BZ substrates and the temperature. As shown in Table 1, the self-oscillating region becomes narrower with an increase in temperature. This result indicates that the self-oscillation behavior is extremely affected by the concentration of NaBrO_3_ because the higher concentration of NaBrO_3_ induced the increase in the size of the polymer aggregation. That is because the initial concentration of the NaBrO_3_ exerts influence on the mole fraction of the Ru(bpy)_3_^3+^ moiety in the polymer chain [14,26]. In the case of the VP-based polymer chain, the size of the polymer aggregation in the oxidized state is much larger than in the reduced state originating from the different solubility of the polymer chain [21–23]. This tendency can be explained by the overall process of the BZ reaction based on the Field-koros-Noyes (FKN) mechanism [27–32]. According to the FKN mechanism, the BZ reaction is divided into three main processes: consumption of Br^−^ ions (process A), autocatalytic formation of HBrO_2_ (process B), and formation of Br^−^ ions (process C).

A $${{\text{BrO}}_{3}}^{-}+2{\text{Br}}^{-}+3{\text{H}}^{+}\to 3\text{HOBr}$$

B $${{\text{BrO}}_{3}}^{-}+{\text{HBrO}}_{2}+2\text{Mred}+3{\text{H}}^{+}\to 2{\text{HBrO}}_{2}+2\text{Mox}+{\text{H}}_{2}\text{O}$$

C $$2\text{Mox}+\text{MA}+\text{BrMA}\to f{\text{Br}}^{-}+2\text{Mred}+\text{other products}$$

In processes B and C, the Ru(bpy)_3_ works as the catalyst: the reduced Ru(bpy)_3_ is oxidized (process B), and the oxidized one is reduced (process C). As the initial concentration of the NaBrO_3_ increases, the mole fraction of the Ru(bpy)_3_^3+^ moiety in the polymer chain increases in accordance with the FKN mechanism. With increasing the Ru(bpy)_3_^3+^ moiety in the polymer chain, the size of the polymer aggregation increases in the oxidized state. That is because the VP-based polymer chain in the oxidized state has lower solubility, compared with the reduced state [21–23]. As shown in Figure 3, when the temperature increases, the period of self-oscillation decreases. As a result, in the high temperature condition, the change in the polymer aggregation state does not synchronize with the BZ reaction because the disaggregation speed of the polymer chains is much slower than the aggregation speed. Therefore, as shown in Table 1, the self-oscillating region becomes narrower especially at the high temperature in the region of the high concentration of NaBrO_3_. This tendency is the same as the self-oscillating behavior of the AMPS-containing polymer chain [12–16]. Moreover, as shown in Table 2, the self-oscillating region becomes wider compared with that shown in Table 1. With an increase in the concentration of the MA, the solubility of the polymer chain in the reduced state increases because the high concentration of the MA advanced the process C. Therefore, the dissociating force of the polymer aggregation in the reduced state increases. As a result, the high concentration of MA increases the self-oscillating region. In addition, as shown in Table 3, no transmittance self-oscillation is observed in the condition of [HNO_3_] = 0.1 M. That is because whether the BZ reaction occurs, is dependent on the concentration of the H^+^, *i.e.*, the BZ reaction has the threshold of the concentration of H^+^ because the H^+^ has a very important role in the BZ reaction as shown in the FKN mechanism.

**Figure 4:**
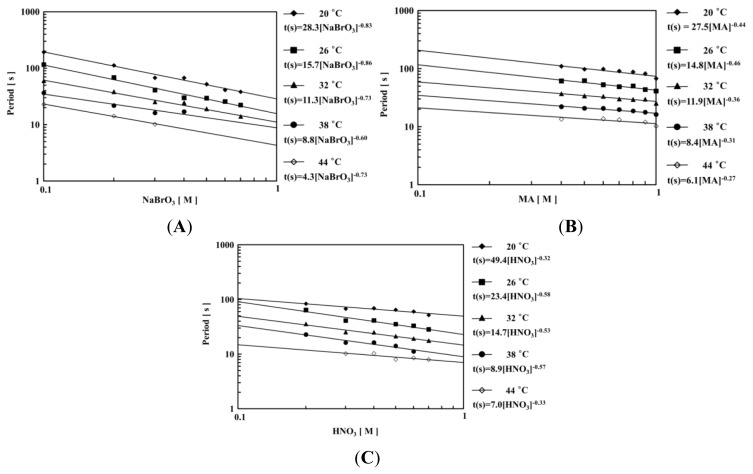
The dependence of the period as a function of temperatures (20–44 °C) and the one BZ substrate under the fixed condition of of (**A**) [MA] = 0.1 M, [HNO_3_] = 0.3 M; (**B**) [NaBrO_3_] = 0.3 M, [HNO_3_] = 0.3 M; (**C**) [NaBrO_3_] = 0.3 M, [MA] = 0.1 M.

Figure 4 shows the logarithmic plots of the period against the initial concentration of one substrate under fixed concentrations of the other two BZ substrates at constant temperatures (*T* = 20–44 °C). As shown in Figure 4, all the logarithmic plots showed a good linear relationship. Therefore, the period *t*(s) of the transmittance self-oscillation can be expressed as *a*[substrate]*^b^* where *a* and *b* are the experimental constants and brackets assign the initial concentration. Moreover, as shown in Figure 4, the period dependence on the concentrations of the BZ substrates has a different aspect as poly(NIPAAm-*co*-Ru(bpy)_3_) gel [33]. In the case of the NIPAA-based polymer gel, the period of the self-oscillation increases with the increase of the concentration of nitric acid. However, in general, the collision frequencies among the BZ substrates increase with increasing temperature. Therefore, we consider that the relationship between the period and the concentration of HNO_3_ for the nonthermoresponsive self-oscillating polymer chain appears to be more natural.

## 3. Materials and Methods

### 3.1. Polymerization

The polymer chain was prepared as follows: 0.5 g of ruthenium(4-vinyl-4′-methyl-2,2′-bipylridine)bis(2,2′-bipyridine)bis(hexafluorophosphate) (Ru(bpy)_3_) as a metal catalyst for the BZ reaction, 9.5 g of vinylpyrrolidone (VP) and 0.35 g of 2,2′-azobis(isobutyronitrile) (AIBN) as an initiator were dissolved in the methanol solution (31 g) under total monomer concentration of 20 wt%. These polymerizations were carried out at 60 °C for 24 h *in vacuo*. These resulting reaction mixtures were dialyzed against graded series of water/methanol mixtures, for 1 day each in 0, 25, 50, 75, and 100 wt% of water, and then freeze-dried.

### 3.2. Measurement of Optical Transmittance Self-Oscillations

The poly(VP-co-Ru(bpy)_3_) solutions were prepared by dissolving the polymer (0.5 wt%) into an aqueous solution containing the three BZ substrates (malonic acid (MA) and sodium bromate (NaBrO_3_), nitric acid (HNO_3_)). The measurements of the transmittance self-oscillation were carried out with a spectrophotometer (JASCO, Model V-630) equipped with magnetic stirrers and a thermostatic controller. The self-oscillations of the polymer solutions were measured under constant temperature and stirring. In order to detect the transmittance change which is based on the autonomous transmittance change, 570-nm wavelength was used because 570-nm wavelength is the isosbestic point of the reduced and oxidized state of the Ru(bpy)_3_ moiety. The time course of the transmittance at 570-nm was monitored by a spectrophotometer.

## 4. Conclutions

We elucidated the activation energy of the aggregation–disaggregation self-oscillation for the polymer chain in the wide range of the three BZ substrate concentrations in a wide temperature range. The activation energy of the self-oscillation is hardly affected by the concentrations of the three BZ substrates. In addition, the activation energy of the self-oscillation is almost the same value as the NIPAA-based self-oscillating gel below the LCST and the normal BZ reaction, i.e. not including the self-oscillating polymer system.

## Figures and Tables

**Table 1:** Phase diagram of the self-oscillating region given by the change in the initial condition of the one BZ substrate and temperature under fixed concentrations of [MA] = 0.1 M, [HNO_3_] = 0.3 M.

Temperature	Concentration of NaBrO_3_						

	0.1 M	0.2 M	0.3 M	0.4 M	0.5 M	0.6 M	0.7 M
20 °C	○	○	○	○	○	○	○
26 °C	○	○	○	○	○	○	○
32 °C	○	○	○	○	○	×	○
38 °C	○	○	○	○	×	×	×
44 °C	○	○	○	×	×	×	×

**Table 2:** Phase diagram of the self-oscillating region given by the change in the initial condition of the one BZ substrate and temperature under fixed concentrations of [NaBrO_3_] = 0.3 M, [HNO_3_] = 0.3 M.

Temperature	Concentration of MA						

	0.04 M	0.05 M	0.06 M	0.07 M	0.08 M	0.09 M	0.10 M
20 °C	○	○	○	○	○	○	○
26 °C	○	○	○	○	○	○	○
32 °C	○	○	○	○	○	○	○
38 °C	○	○	○	○	○	○	○
44 °C	○	×	○	○	×	○	○

**Table 3:** Phase diagram of the self-oscillating region given by the change in the initial condition of the one BZ substrate and temperature under fixed concentrations of [NaBrO_3_] = 0.3 M, [MA] = 0.1 M. A cross means that no oscillation occurs.

Temperature	Concentration of MA						

	0.1 M	0.2 M	0.3 M	0.4 M	0.5 M	0.6 M	0.7 M
20 °C	×	○	○	○	○	○	○
26 °C	×	○	○	○	○	○	○
32 °C	×	○	○	○	○	○	○
38 °C	×	○	○	○	○	○	×
44 °C	×	×	○	○	○	○	○
